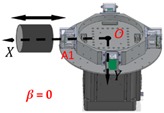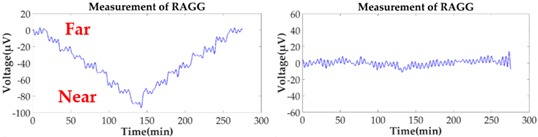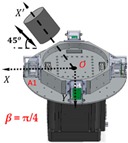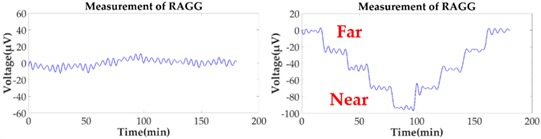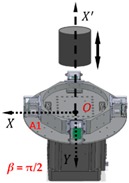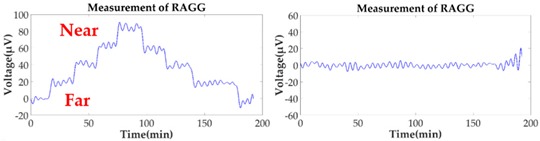# Scale Factor Calibration for a Rotating Accelerometer Gravity Gradiometer

**DOI:** 10.3390/s18124386

**Published:** 2018-12-11

**Authors:** Zhongguang Deng, Chenyuan Hu, Xiangqing Huang, Wenjie Wu, Fangjing Hu, Huafeng Liu, Liangcheng Tu

**Affiliations:** 1 MOE Key Laboratory of Fundamental Physical Quantities Measurement & Hubei Key Laboratory of Gravitation and Quantum Physics, PGMF and School of Physics, Huazhong University of Science and Technology, Wuhan 430074, China; dzg_109@hust.edu.cn (Z.D.); wjwu@hust.edu.cn (W.W.); fangjing_hu@hust.edu.cn (F.H.); huafengliu@hust.edu.cn (H.L.); 2 TianQin Research Center for Gravitational Physics and School of Physics and Astronomy, Sun Yat-Sen University (Zhuhai Campus), Zhuhai 519082, China; huangxq59@sysu.edu.cn; 3 Institute of Geophysics and PGMF, Huazhong University of Science and Technology, Wuhan 430074, China

**Keywords:** gravity gradient measurement, rotating accelerometer gravity gradiometer, accelerometer, static-platform calibration, scale factor

## Abstract

Rotating Accelerometer Gravity Gradiometers (RAGGs) play a significant role in applications such as resource exploration and gravity aided navigation. Scale factor calibration is an essential procedure for RAGG instruments before being used. In this paper, we propose a calibration system for a gravity gradiometer to obtain the scale factor effectively, even when there are mass disturbance surroundings. In this system, four metal spring-based accelerometers with a good consistency are orthogonally assembled onto a rotary table to measure the spatial variation of the gravity gradient. By changing the approaching pattern of the reference gravity gradient excitation object, the calibration results are generated. Experimental results show that the proposed method can efficiently and repetitively detect a gravity gradient excitation mass weighing 260 kg within a range of 1.6 m and the scale factor of RAGG can be obtained as (5.4 ± 0.2) E/μV, which is consistent with the theoretical simulation. Error analyses reveal that the performance of the proposed calibration scheme is mainly limited by positioning error of the excitation and can be improved by applying higher accuracy position rails. Furthermore, the RAGG is expected to perform more efficiently and reliably in field tests in the future.

## 1. Introduction

The gravity gradient signal is very important data of Earth. Gravity gradient measurements have attracted considerable attention in the fields of resource exploration, gravity aided navigation, etc. [1,2,3]. Hungarian physicist Roland von Eötvös invented the first gravity gradiometer using a torsional pendulum in 1890, and it was applied for oil and gas field exploration. However, it took several hours to measure a single point and required a quiet measure environment [4]. The first gravity gradient measurement based on a moving platform was accomplished through design, manufacture, and testing in 1982, by Bell Aerospace Inc. (Buffalo, NY, USA) [5,6]. In the 1990s, BHP Billiton from Australia cooperated with Lockheed Martin (former Bell Aerospace), and developed the partial gradient component measurement system for geological exploration using the Rotating Accelerometer Gravity Gradiometer (RAGG) technology. It was completed in 1997 and named the Falcon^TM^ Airborne Gravity Gradiometer (AGG), with a noise performance of 3.3 E achieved in a 0.18 Hz bandwidth and it came into service after a two-year flight test [7,8]. Moreover, Lockheed Martin upgraded the system from a marine Full Tensor Gradiometer to an air Full Tensor Gradiometer named Air-FTG^TM^, with noise power densities of 7–8 E^2^·km in a Cassna C208 and 5–6 E^2^·km in a Basler BT-67. Since 2014, it has flown a two million kilometer test-line, and has shown lots of advantages and excellent success in navigation and commercial applications [9,10]. Starting from the 1990s, rapid progress has been made in atomic interference, superconducting, and other technologies. Atomic interferometer gravity gradiometers (such as Stanford AI), superconducting gravity gradiometers (such as VK1), and other gravity gradiometers based on late-model technologies have come into public sight [11,12,13]. 

RAGG is one of the few airborne gravity gradiometers which has been applied for navigation and resource exploration. E.H. Metzger, who led the first RAGG development, reported the status of the RAGG development program and gave the instrument structures and self-generated noise, including thermal noise and electronic noise. He also reported that the RAGG output fluctuation was less than 2 E in a stability experiment of 18 h and the instrument bias trend was about 2.2 × 10^−3^ E/h [14,15,16]. However, the scale factor calibration had not been reported and the output unit of RAGG was directed to E. Hofmeyer et al., who focused on the intrinsic noise of the RAGGs and proved an achievable sensitivity below 3 E/√Hz for stationary measurements using the eight-accelerometer gravity gradiometer and the performance has been improved through optimization in the gradiometer measurement [17,18,19]. Although the sensitivity has been tested and calculated by E/√Hz instead of V/√Hz, the scale factor calibration of RAGG has still not been released. Cai et al. calculated and simulated a calibration method of an RAGG using centrifugal gradients, and provided detailed procedures and mathematical formulations for calibrating scale factors and other parameters in their model [20]. However, the particular and detailed RAGG calibration method needs to be further verified in experiments.

In this paper, we propose a calibration system of a gravity gradiometer to obtain the scale factor of RAGG. In this calibration system, RAGG measures the spatial variation of the gravity gradient caused by the approaching reference mass body. In order to alleviate the influence of surrounding masses and the human disturbance, we change the direction of the gravity gradient excitation move towards and away from RAGG, and the calibration results can finally be demodulated. The process shows the repeatability of the scale factor calibration and obtains a stable scale factor. Additionally, the theoretical analysis results are highly consistent with the experimental results.

## 2. Principle

By the law of superposition, the gravitational field potential can be generalized using the concept of mass density, as shown in Equation (1):

(1) $$V\left(r\right)=\int_{V}\frac{G\rho \left(r-r^{\prime }\right)}{\left|r-r^{\prime }\right|}dV,$$

where ***r*** denotes the location where the potential is determined, ***r*′** indicates the location of differential volume element d*V*, and *G* is the gravitational constant.

The gravitational acceleration g is the first order derivative of the spatial gravitational potential, and the gravity gradient is the second order derivative of the spatial gravitational potential. The spatial gravity gradient tensor is given by

(2) $$\Gamma =\left(\begin{array}{l} \frac{\partial g_{ox}}{\partial x}\frac{\partial g_{oy}}{\partial x}\frac{\partial g_{oz}}{\partial x}\\ \frac{\partial g_{ox}}{\partial y}\frac{\partial g_{oy}}{\partial y}\frac{\partial g_{oz}}{\partial y}\\ \frac{\partial g_{ox}}{\partial z}\frac{\partial g_{oy}}{\partial z}\frac{\partial g_{oz}}{\partial z} \end{array}\right)=\left(\begin{array}{l} \frac{\partial^{2}V}{\partial x^{2}}\frac{\partial^{2}V}{\partial x\partial y}\frac{\partial^{2}V}{\partial x\partial z}\\ \frac{\partial^{2}V}{\partial x\partial y}\frac{\partial^{2}V}{\partial y^{2}}\frac{\partial^{2}V}{\partial y\partial z}\\ \frac{\partial^{2}V}{\partial x\partial z}\frac{\partial^{2}V}{\partial y\partial z}\frac{\partial^{2}V}{\partial z^{2}} \end{array}\right)=\left(\begin{array}{l} \Gamma_{xx}{\;\Gamma }_{xy}{\;\Gamma }_{xz}\\ \Gamma_{xy}{\;\Gamma }_{yy}{\;\Gamma }_{yz}\\ \Gamma_{xz}{\;\Gamma }_{yz}{\;\Gamma }_{zz} \end{array}\right)$$

**Figure 1:**
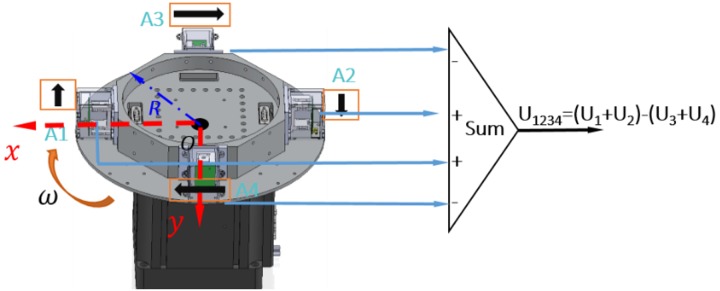
Schematic diagram of the mechanical modulation of the RAGG.

Due to the continuity of the derivatives of the potential, and considering Poisson’s field equation, the gravity tensor is symmetric and its trace is zero. Generally, the gravity gradient signal is extremely weak, whose unit, 1 E, is defined as the gravity difference of approximately 0.102 ng (1 g ≈ 9.8 m/s^2^) between two mass bodies with a distance of 1 m. The RAGG employs mechanical rotation modulation and synchronous electrical demodulation to extract the gravity gradient signal as a lock-in amplifier, as shown in Figure 1. In order to measure the gravity gradient at point *O*, the four accelerometers are assembled onto a disc whose center is point *O*. The directions of the sensitive axes of the accelerometers, which the black arrows indicate, are along the tangent of the disk in a clockwise manner. Therefore, the sensitive axes of the two adjacent accelerometers are orthogonal to each other. The disk is then driven by a precision motor at a constant angular rate, ω.

The output model of the accelerometers that we used is $U=K_{I}a_{I}$, where U,$K_{I}$,$a_{I}$, are the output voltage, scale factor, and acceleration along the direction of the sensitive axis of the accelerometer, respectively. By expanding the Taylor series of the $i^{th}$ accelerometer (i=1,2,3,4) at point *O*, the summed output of the four accelerometers can be expressed by Equation (3):

(3) $$U_{1234}=-K_{Id}g_{ox}\sin\omega t+K_{Id}g_{oy}\cos\omega t+\;K_{Id}\frac{\partial \omega }{\partial t}R+\frac{1}{2}K_{Is}\left(\Gamma_{yy}-\Gamma_{xx}\right)R\sin2\omega t+K_{Is}\Gamma_{xy}R\cos2\omega t,$$

where $K_{Id}=\left(K_{I1}+K_{I2}\right)-\left(K_{I3}+K_{I4}\right)$, $K_{Is}=K_{I1}+K_{I2}+K_{I3}+K_{I4}$. $g_{ox},g_{oy}$ are the component of the acceleration of gravity at the *x* and *y* axis, respectively, and $\frac{\partial \omega }{\partial t}$ is the rotation angular acceleration of the rotary table.

According to Equation (1), the gravity gradient signal at point *O*, $\Gamma_{yy}-\Gamma_{xx}\;,$ and $\Gamma_{xy}$, can be modulated at the 2ω frequency domain, which is double the rotational frequency, successfully. By demodulating $U_{1234}$ using the reference signals sin2ωt and cos2ωt, the gravity gradient tensor component can be obtained through a low-pass filter. From the frequency domain, the horizontal component of the gravitational acceleration will couple to the measured value of the gravity gradient basis of the trigonometric function. Likewise, the 2ω component of the rotary speed will contribute to the output result of the gravity gradient and the angular rate noise is a relatively diminutive error. Furthermore, the static surrounding masses cause a gravity gradient, which will also contribute to zero bias of the RAGG output. Since the relative variation of the gravity gradient, rather than the absolute measurement, is measured by RAGG, little consideration is taken in these zero bias issues in the calibration experiments. 

**Figure 2:**
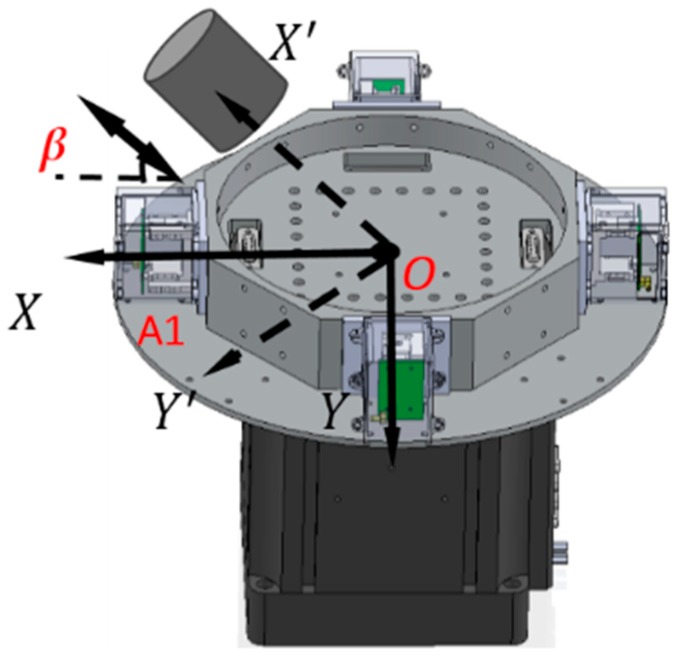
Schematic diagram of the calibration experiments.

Assuming that the RAGG is put in the coordinate *O-XYZ* (coordinate B) and the direction of baseline *O*-A1 is along the *X* axis, RAGG measures the gravity gradient that is dependent on coordinate *O-XYZ.* At the same time, creating coordinate *O-X′Y′Z′* (coordinate A) for the movement pattern of the reference mass object, and letting *Z′* and *Z* axis coincide, the angle between *X′* and *X* axis is *β*, as shown in Figure 2.

Then, the gravity gradient tensor $\Gamma^{B}$ is obtained by using the tensor transformation principle:

(4) $$\Gamma^{B}=[\begin{array}{ccc} \Gamma_{XX} & \Gamma_{XY} & \Gamma_{XZ}\\ \Gamma_{YX} & \Gamma_{YY} & \Gamma_{YZ}\\ \Gamma_{ZX} & \Gamma_{ZY} & \Gamma_{ZZ} \end{array}]=C_{A}^{B}\Gamma^{A}C_{B}^{A}=C_{A}^{B}\;[\begin{array}{ccc} \Gamma_{X^{\prime }X^{\prime }} & \Gamma_{X^{\prime }Y^{\prime }} & \Gamma_{X^{\prime }Z^{\prime }}\\ \Gamma_{Y^{\prime }X^{\prime }} & \Gamma_{Y^{\prime }Y^{\prime }} & \Gamma_{Y^{\prime }Z^{\prime }}\\ \Gamma_{Z^{\prime }X^{\prime }} & \Gamma_{Z^{\prime }Y^{\prime }} & \Gamma_{Z^{\prime }Z^{\prime }} \end{array}]C_{B}^{A},$$

where,$C_{A}^{B}$ is the direction cosine matrix from coordinate A to B, as:

(5) $$C_{A}^{B}=\left[\begin{array}{ccc} \cos\beta & \sin\beta & 0\\ -\sin\beta & \cos\beta & 0\\ 0 & 0 & 1 \end{array}\right],\;C_{B}^{A}={\left(C_{B}^{A}\right)}^{\prime }$$

So, the gravity gradient that the mass body cylinder excited in the *O-X′Y′Z′* can be measured by the RAGG instrument described as the gravity gradient in the *O-XYZ*, given by:

(6) $$\left\{ \begin{array}{c} \;\Gamma_{XX}=\Gamma_{X^{\prime }X^{\prime }}\cos^{2}\beta +2\Gamma_{X^{\prime }Y^{\prime }}\sin\beta \cos\beta +\Gamma_{Y^{\prime }Y^{\prime }}\sin^{2}\beta \\ \Gamma_{XY}=(\Gamma_{Y^{\prime }Y^{\prime }}-\Gamma_{X^{\prime }X^{\prime }})\sin\beta \cos\beta -\Gamma_{X^{\prime }Y^{\prime }}(\sin^{2}\beta -\cos^{2}\beta ).\\ \Gamma_{YY}=\Gamma_{Y^{\prime }Y^{\prime }}\cos^{2}\beta -2\Gamma_{X^{\prime }Y^{\prime }}\sin\beta \cos\beta +\Gamma_{X^{\prime }X^{\prime }}\sin^{2}\beta \end{array}\right.$$

When the direction of the reference mass object movement has an angle *β* towards the RAGG’s baseline *O*-A1, the RAGG’s output can be expressed by:

(7) $$U_{1234}=\frac{1}{2}K_{Is}\left[(\Gamma_{Y^{\prime }Y^{\prime }}-\Gamma_{X^{\prime }X^{\prime }}\right)\cos2\beta -2\Gamma_{X^{\prime }Y^{\prime }}\sin2\beta ]R\sin2\omega t\;+\frac{1}{2}K_{Is}[(\Gamma_{Y^{\prime }Y^{\prime }}-\Gamma_{X^{\prime }X^{\prime }})\sin2\beta +2\Gamma_{X^{\prime }Y^{\prime }}\cos2\beta ]R\cos2\omega t.$$

However, in the coordinate *O-X′Y′Z′*, the gravity gradient that the mass body moves towards and away from RAGG always stays the same. From Equation (2), the gravity gradient tensor $\Gamma_{X^{\prime }Y^{\prime }}$ is equal to zero and $\Gamma_{Y^{\prime }Y^{\prime }}-\Gamma_{X^{\prime }X^{\prime }}$ is non-zero, which can be seen as the excitation signal when the excitation pattern is following the *X′*-axis. However, in the coordinate *O-XYZ*, the RAGG measurement output varies with sin2β and cos2β. For example, by demodulating $U_{1234}$ using the reference signals, the calibration signal appears in the sin2ωt demodulation when β=0; however, it appears in the cosωt demodulation when β=π/4. Additionally, the phenomenon for which the results vary with sin2β and cos2β of a 50 E step change is shown in Table 1.

When the gravity gradiometer is calibrated, we can set the angle β=0 first, and get a calibration step signal. Secondly, by changing the angle to β=π/4 and β=π/2, the influence of masses of the surrounding can be decreased because the influence will not change along with the angle β during the calibration. Therefore, the masses influenced by the surrounding, such as human disturbance, can be decreased by multiple measurements of the reference object with a different angle β.

## 3. Experimental Results

**Figure 3:**
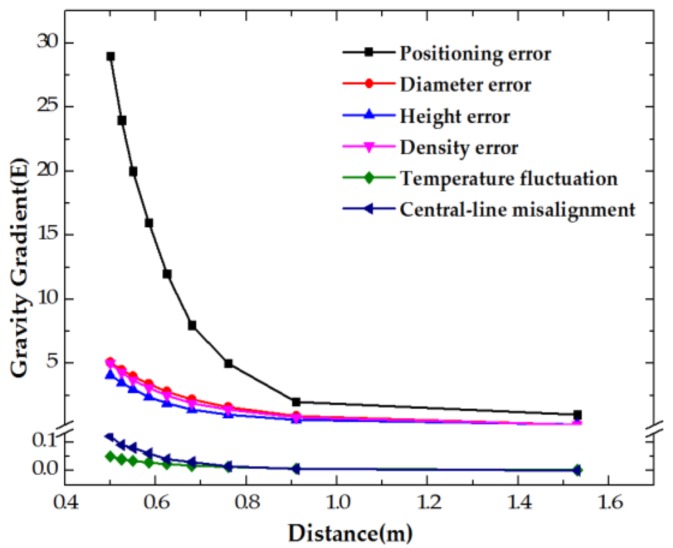
Theoretical calculation curve of gravity gradient.

In the scale factor calibration experiment, we chose lead as the mass body material due to its high density and relatively cheap price. In order to get a gravity gradient where the excitation varies from ~0 E to ~500 E within the distance of ~0.5 m to ~1.6 m, a lead cylinder, whose height is 33 cm and diameter is 32 cm, was used in the experiment. However, some system errors, such as volume error (especially, to error of height and diameter) and density error of the lead cylinder, accuracy of positioning, central line of cylinder misalignment to point *O*, and temperature fluctuation to change the volume (the coefficient of thermal expansion of lead, 2.9 × 10^−5^/°C), should be discussed. Moreover, these system errors are varied from one location to another. Finally, the processing error of each term and its contributions to the gravity gradient are shown in Table 2 and Figure 3.

**Figure 4:**
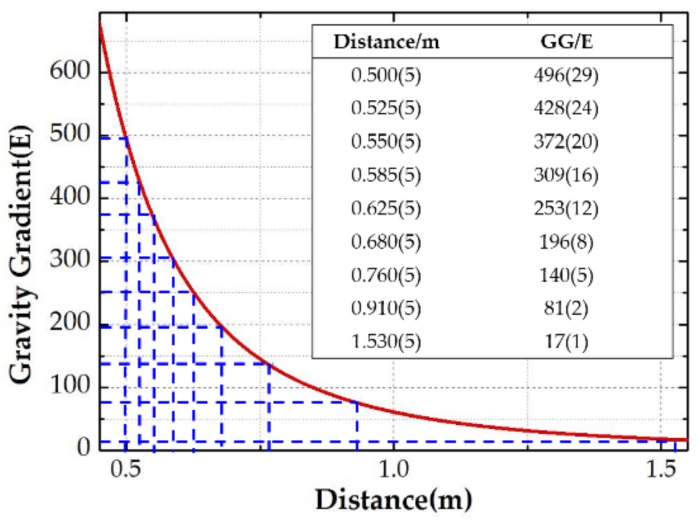
Theoretical calculation curve of gravity gradient.

As shown in Figure 3, the positioning error of the lead cylinder is the main error source. Furthermore, the gravity gradient $\Gamma_{Y^{\prime }Y^{\prime }}-\Gamma_{X^{\prime }X^{\prime }}$ that the lead cylinder excited in the coordinate *O-X′Y′Z′* and the positioning error in each location are calculated, as shown in Figure 4. 

**Figure 5:**
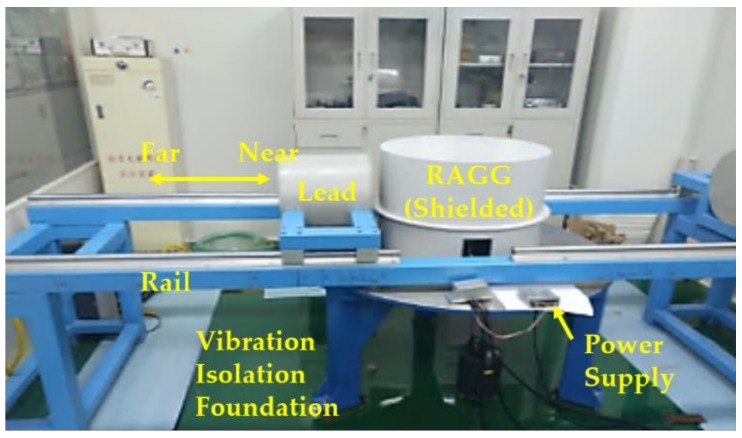
Experiment setup for RAGG’s scale factor calibration.

The calibration experiment setup was built in a laboratory, where the temperature was kept at 23 ± 1 °C, and the setup was shielded to avoid electromagnetic interference. The gravity gradient instrument was put on the point *O*. A lead cylinder weighing 260 kg, acting as the gravity gradient excitation, was built and mounted on a rail. In order to eliminate the disturbance of ground vibration with someone passing through, the RAGG was put on the vibration isolation foundation and the lead cylinder was suspended along the rail whose supporting feet were outside the vibration isolation foundation, as shown in Figure 5.

**Figure 6:**
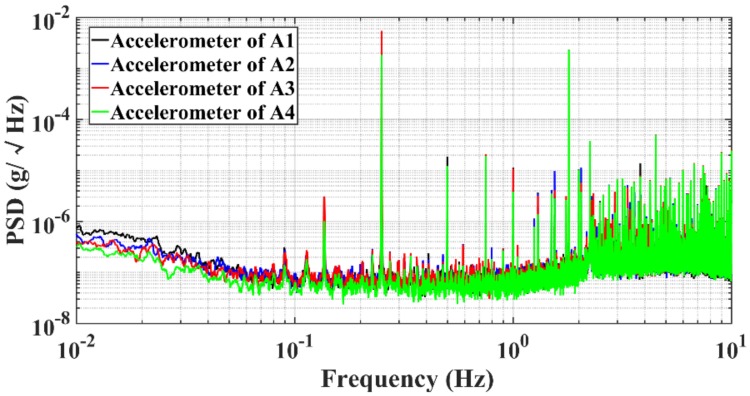
The noise floor of four accelerometers in a rotating measurement.

The four accelerometers were then assembled onto the rotary motion table for the measurement of the gravity gradient. The rotating frequency of the rotary table was 0.25 Hz. The base line was from the proof mass (PM) of the accelerometers to point *O*, and the radius of the rotary disc was 200 mm. It can be seen from Figure 6 that the noise floor of the four accelerometers was ~50 ng/√Hz@0.25 Hz.

**Figure 7:**
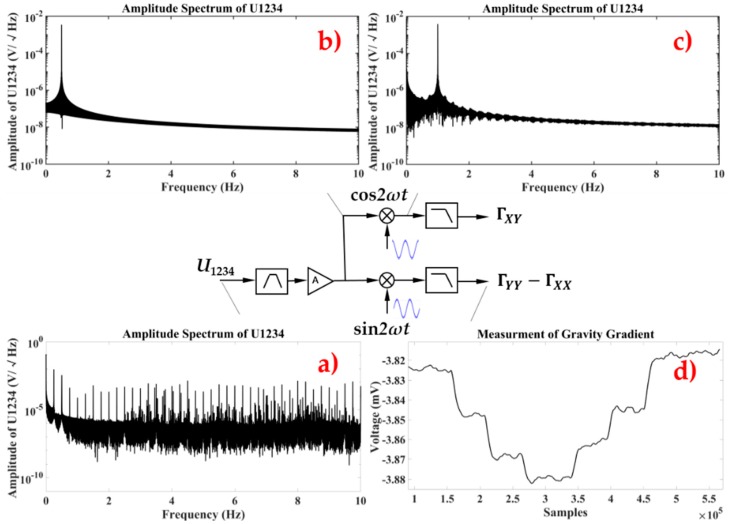
The procedure for gravity gradient data extracting. (**a**) Power Spectrum Density (PSD) of U_1234_. (**b**) PSD of U_1234_ after band-pass filter of 0.5 Hz. (**c**) PSD of U_1234_ after demodulating. (**d**) Gravity gradient signal was extracted through an averaging of 120 s.

The data extracting of the gravity gradient measurement at point *O* is shown in Figure 7. After a band-pass and amplifying stage, $\Gamma_{yy}-\Gamma_{xx}$ and $\Gamma_{xy}$ can be extracted by demodulation, with reference signals of sin2ωt and cos2ωt, respectively.

Without the loss of generality, the lead cylinder was pushed towards and away from the RAGG with various distances of 153 cm, 91 cm, 76 cm, …, 52.5 cm, and 50 cm along the *X*-axis. For the repeatability to measure the scale factor of the RAGG, the movement direction was changed by the *Y*-axis and 45 degrees line in the first quadrant. A step change of 50 E in the β=0, and a 100 E change in the β=π/4 or β=π/2, were designed. The measured results of the gravity gradient at point *O* are shown in Table 3. 

As is shown in Table 3, this RAGG system is able to detect a gravity gradient excitation (260 kg) within a nearly 1.6 m range. From Equation (2), the gravity gradient tensor $\Gamma_{X^{\prime }Y^{\prime }}$ is equal to zero and $\Gamma_{Y^{\prime }Y^{\prime }}-\Gamma_{X^{\prime }X^{\prime }}$ is non-zero, which can be an excitation signal when the excitation pattern follows the *X′*-axis, which is shown in Table 1 when β=0. From Equation (6), when β=π/4, the demodulated result has the calibration curve that displays the same trend as β=0. Additionally, for β=π/2, the sin2ωt demodulated result has the calibration curve which displays the opposite trend as β=0, and it is matched in the theoretical analysis, as shown in Table 1.

**Figure 8:**
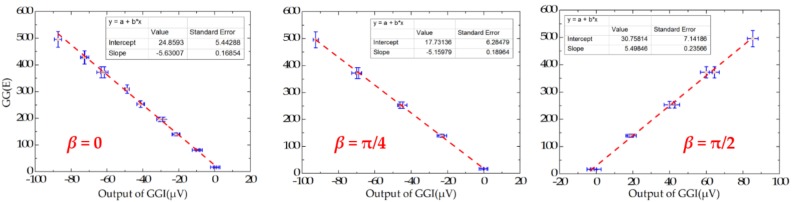
Fitting curve of scale factor of static-platform state calibration of the RAGG.

In order to analyze the scale factor of RAGG, the calibration step results are line-fitted with the X and Y error bar, as is shown in Figure 8. The scale factor of RAGG is (5.6 ± 0.2) E/μV, (5.2 ± 0.2) E/μV, and (5.5 ± 0.2) E/μV, while the angles are β=0, β=π/4, and β=π/2, respectively. Finally, it is (5.4 ± 0.2) E/μV through the error analysis. Since the accuracy of the results is mainly dependent on the lead cylinder positioning error, there is still great potential for improvement in the calibration experiment.

**Figure 9:**
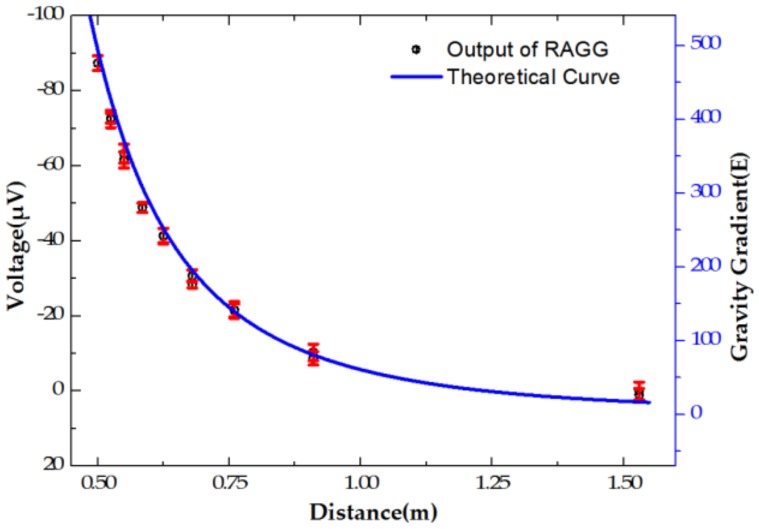
Measurement results versus theoretical calculation.

In order to analyze the accuracy of the gravity gradient measurement, the excitation motion is adjusted until the tensor $\Gamma_{xy}$ remains instant. Figure 9 shows the measured results by changing the excitation distance of from 50 to 155 cm, and compares them with theoretical calculations. The curve shows a good consistency when repeating the excitation pattern approaching/retreating point *O*. 

## 4. Conclusions

In this paper, we proposed a static-platform calibration system of a gravity gradiometer to obtain a stable scale factor of the RAGG. The gravity gradient signal extraction technology, based on the rotation accelerometers by lock-in amplifier technology with mechanical modulation and electrical demodulation, was studied. Then, a gravity gradient based on a field verification platform for gravity gradiometer calibration was built. The direction of the gravity gradient excitation moving towards and away from RAGG was changed to alleviate the effect of the surroundings, and finally, the calibration output showed different results by demodulating. The process demonstrated the repeatability of the scale factor calibration and obtained a stable scale factor of RAGG, (5.4 ± 0.2) E/μV. The theoretical analysis results were highly consistent with the experimental results.

We have established an experimental setup of RAGG for calibration use, and the experimental results were mainly limited by the positioning error of the mass body of excitation and could be further improved by using a higher accuracy position horizontal guide rail. Furthermore, we acknowledge that the scale factor calibration will be improved by considering a more accurate gravity gradient excitation model including high order effects, a spherical lead ball, a higher precision accelerometer, multiple independent RAGG measurements, and so on.

## Figures and Tables

**Table 1:** Simulation results of RAGG with different angle β.

Different Angle *β*	Demodulated by sin2*ωt*	Demodulated by cos2*ωt*
β=0	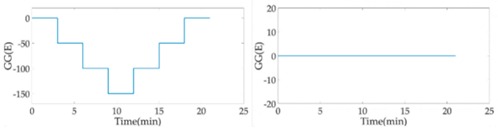	
β=π/4	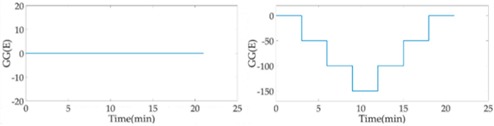	
β=π/2	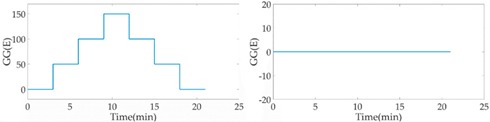	

**Table 2:** Processing system error of the calibration experiment setup.

System Error Terms	Processing Error	System Error Terms	Processing Error
Height of cylinder	±2 mm	Positioning along rail	±5 mm
Diameter of cylinder	±2 mm	Temperature fluctuation	±1 °C
Density of Lead	*δρ/ρ* ~ 1%	Central line misalignment	±5 mm

**Table 3:** Calibration of RAGG with excitation at different toward-away direction patterns.

Different Toward-Away Directions	Demodulated by sin2*ωt*	Demodulated by cos2*ωt*